# Fresh osteochondral allograft transplantation for knee full-thickness articular cartilage lesions using femoral head of living donors: short-term results

**DOI:** 10.1007/s00402-024-05413-3

**Published:** 2024-07-15

**Authors:** Hesham Ossama Soubih, Ahmed M. Al-Saed, Sherif A. El Ghazaly, Mohamed H. Sobhy, Muhammad Elsayed Kamel, Wessam Fakhry Ebied, Haitham K. Haroun

**Affiliations:** Orthopedic Department, Faculty of Medicine, Ain Shams University, Cairo Governorate, Egypt

**Keywords:** Osteochondral allografts, Femoral heads, Living donors

## Abstract

**Background:**

Fresh osteochondral allograft transplantation is a good treatment option of cartilage defects. However, this treatment option is not available in all countries due to limited graft availability and tissue banks limitations. The purpose of this study is to assess the short term functional and imaging outcomes of fresh osteochondral allograft transplantation in the knee using the femoral head of living donors.

**Hypothesis:**

Fresh osteochondral allografts from the femoral heads of living donors is a valid graft source for management of distal Femur cartilage defects. This technique can improve functional knee scores with good radiological outcomes.

**Study Design:**

Prospective case series.

**Methods:**

Fifteen patients with full thickness cartilage defects of the distal femur underwent osteochondral allograft transplantation from the femoral heads of living donors. Grafts were transplanted by both shell and multiple dowels techniques. The average follow up duration was 18.3 months (range, 12–25 months). Patients were evaluated by Lysholm and International Knee Documentation Committee (IKDC) scores, radiography and MR imaging using Osteochondral Allograft MRI Scoring System (OCAMRISS).

**Results:**

There was a statistically significant improvement (*P* < 0.001) in both Lysholm and IKDC average scores at 6 months and 12 months postoperative. Postoperative MRI was done at an average 6.8 months (range, 5–11 months) postoperative. The mean total OCAMRISS score was 3.4 (range, 1–7). A second look arthroscopy was done in four patients and showed intact articular cartilage in all three patients.

**Conclusion:**

Femoral head of living donors is a valid new source for fresh osteochondral allograft transplantation of knee osteochondral lesions. Short term results showed improvement in clinical assessment scores. Follow up imaging showed graft incorporation and good MRI scores.

## Key message

**What is known about the subject:**

Fresh osteochondral allograft transplantation is a successful treatment option for full thickness cartilage lesions in the knee. Osteochondral allografts are harvested from the knee of young deceased donors. There are limitations regarding the graft availability and postmortem tissue banks.

**What this study adds to existing knowledge:**

In our study, we used fresh osteochondral allografts from the femoral heads of living donors. The donor population is the non-arthritic patients who undergo hip replacement for traumatic neck femur fractures. This will provide a new source for fresh osteochondral allografts in countries who lack postmortem tissue banks. Even with available tissue banks, this provides an additional stable source of grafts which may expand the indications for osteochondral allografts and facilitate preoperative scheduling. To our knowledge, this is the first clinical study that used the femoral head of living donors as a source of fresh osteochondral allografts in the knee.

## Introduction

Focal Articular Cartilage injuries are common as they can be found in more than 50% of knee arthroscopies. Abrasion chondroplasty, microfracture, osteochondral allograft, osteochondral autograft, and autologous chondrocyte implantation are the main available treatment lines [1–6]. Microfracture and drilling are technically easy and short procedures with good short term results. However, the data are insufficient regarding the long term results and the results are less favourable in lesions larger than 4 cm^2^ and patellar lesions [5, 7–9].

Osteochondral autografts(OATs) means harvesting the osteochondral plugs from non weight bearing areas of the patient’s knee in a single stage procedure. Disadvantages include limited donor surface area and donor site morbidity. Results of OATs are dependent on the lesion size. Good to excellent results are reported with lesion size 1 to 4 cm^2^. Poor results are reported with lesion size more than 6 cm^2^ [10]. Autologous Chondrocyte implantation is another treatment option that can restore large chondral injuries. However, it is difficult to restore the subchondral bone and the results are poor with large osteochondral defects [11, 12].

Fresh osteochondral allograft transplantation is a good treatment option because it restores both the hyaline cartilage and subchondral bone in a single stage procedure allowing reconstruction of large complex defects and uncontained lesions. The mid-term and long-term patient-reported outcomes are good with 80% graft survival in five to ten years [13, 14]. There is no donor size morbidity, limitation to the graft size or difficulty to restore the subchondral bone. The allograft survival rate and patient-reported outcomes are comparable to osteochondral autografts [15]. Osteochondral allograft has lower reoperation rate and lower cost compared to autologous chondrocyte implantation [12].

Fresh osteochondral allografts pose several limitations. First limitaion is the cost and availability of post mortem tissue banks. They are unavailable in some countries due to increased cost and religious restrictions [10, 16]. Second limitation, osteochondral allografts have the potential of disease transmission. Graft contamination can result from occult infection of the donor, postmortem invasion of the tissues by gut flora, or contamination during the recovery process [17]. The number of viable chondrocytes starts to decrease after 14 days from the harvest and reaches the least accepted count after 28 days. This leads to the third limitation, a narrow 14 days’ time frame for recipient matching and preoperative preparations [18], leading to a complex preoperative scheduling.

Femoral heads represent an alternative source for fresh osteochondral allografts. Femoral heads can be retrieved from non-arthritic patients who undergo femoral head replacement for femoral neck fractures. This technique carries the theoretical advantages of graft availability, low cost and lower risk of infection. It could present a solution for the problem of large or uncontained cartilage defects in countries lacking postmortem tissue banks and chondrocyte implantation technology. However there is a theoretical concern regarding the graft contour. The femoral head is more convex than the distal femoral condyles. The purpose of this prospective study is to assess the short term functional and imaging outcomes of fresh osteochondral allograft transplantation for knee full-thickness articular cartilage lesions using femoral head of living donors. Our hypothesis is fresh osteochondral allografts from the femoral heads of living donors is a valid graft source for management of distal femur cartilage defects.

## Materials and methods

### Patient selection

This study was approved by our hospital’s Research Ethics Committee (Faculty of Medicine Ain Shams University Research Ethics Committee [FMASU REC] with reference number FWA 000017585). Written informed consents were signed by all participants. Between April 2021 and June 2022, we included patients who had osteochondral allograft transplantation for a symptomatic full thickness cartilage lesion in the distal femur articular surface that failed conservative medical treatment and physiotherapy. We excluded patients with bipolar cartilage lesions, inflammatory arthropathy, body mass index more than 30, untreated limb malalignment or ligament injury and absence of more than 50% of ipsilateral meniscus.

Full thickness cartilage lesions were diagnosed by history, examination and MR images. Radiography was performed to assess subchondral bone and exclude osteoarthritis. Knee arthroscopy was performed in the same operative session to diagnose other knee pathologies, confirm the diagnosis of full thickness cartilage lesion and assess the defect size.

### Graft selection

Fresh osteochondral allografts were taken from the femoral heads of patients undergoing arthroplasty for traumatic femoral neck fractures. Donors were screened preoperatively for viral markers and history of malignancy or collagen disease. We did not perform blood-type or human leukocyte antigen matching as it is not needed in the standard postmortem fresh allografts. Assessment of the femoral head cartilage status included two main steps [19]. First, donors’ hip radiographs were assessed using the Tonnis grading system [20]. Only donor hips with Tonnis grade 0 (No signs of hip arthrosis) were included. Second, gross inspection and taking photos were used to score the femoral head cartilage status according to the International Cartilage Repair Society (ICRS) grading system [5]. Only femoral heads with grade 0 (No cartilage injury) cartilage status were included. Femoral heads were harvested, stored and transplanted under complete aseptic conditions without a sterilization technique to avoid chondrocytes destruction. They were stored at 4 °C in a Lactate Ringer’s solution containing polymyxin B bacitracin antibiotics as the standard postmortem fresh allografts [21–25].

Fresh osteochondral allografts transplantation started in university based centers. The allografts were transplanted within a week from the harvest. This was favorable regarding the number of viable chondrocytes [8]. When allografts became widely available through uniform cartilage banking, safety concerns resulted in guidelines with a minimum 14 days serologic and bacteriologic test panels. The number of viable chondrocytes start to decrease after 14 days. The minimum number of viable chondrocytes sufficient for transplantation is thought to be at 28 up to 35 days. So, usually by the time of transplantation, the number of viable chondrocytes has decreased due to apoptosis [8, 19]. In our study, being a university based research study, the allograft was transplanted within average 6 days from the harvest. Since allografts from living donors have lower risk of infection, there is a possibility these grafts can have shorter duration test panel. This can be favorable for chondrocytes viability.

### Operative technique

**Fig. 1:**
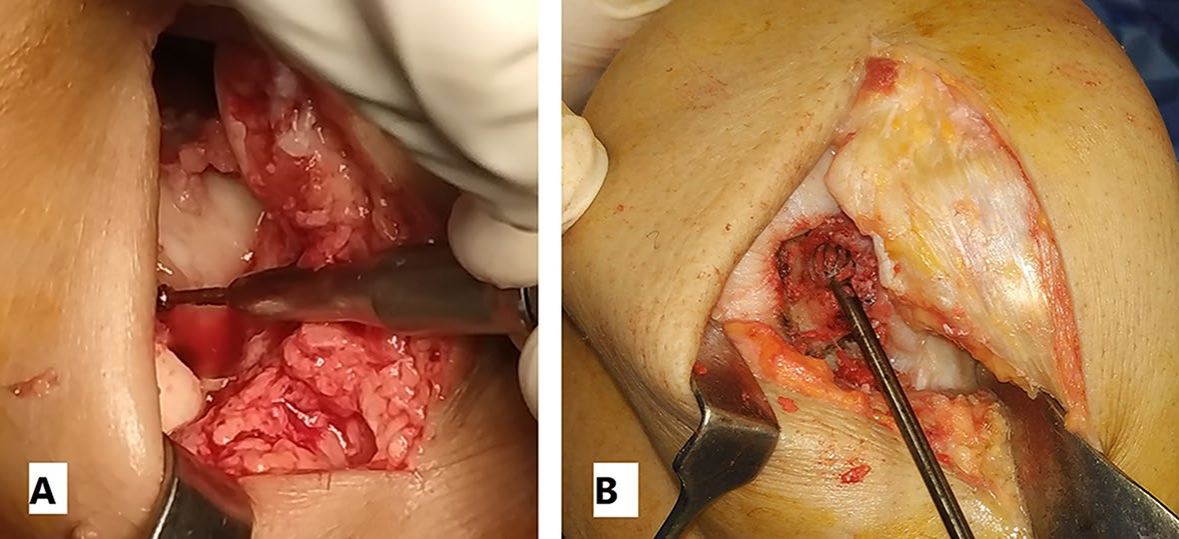
Subchondral bone was cut to the depth of 6–8 mm by a burr (**A**: No. 7 patient, **B**: No. 3 patient)

**Fig. 2:**
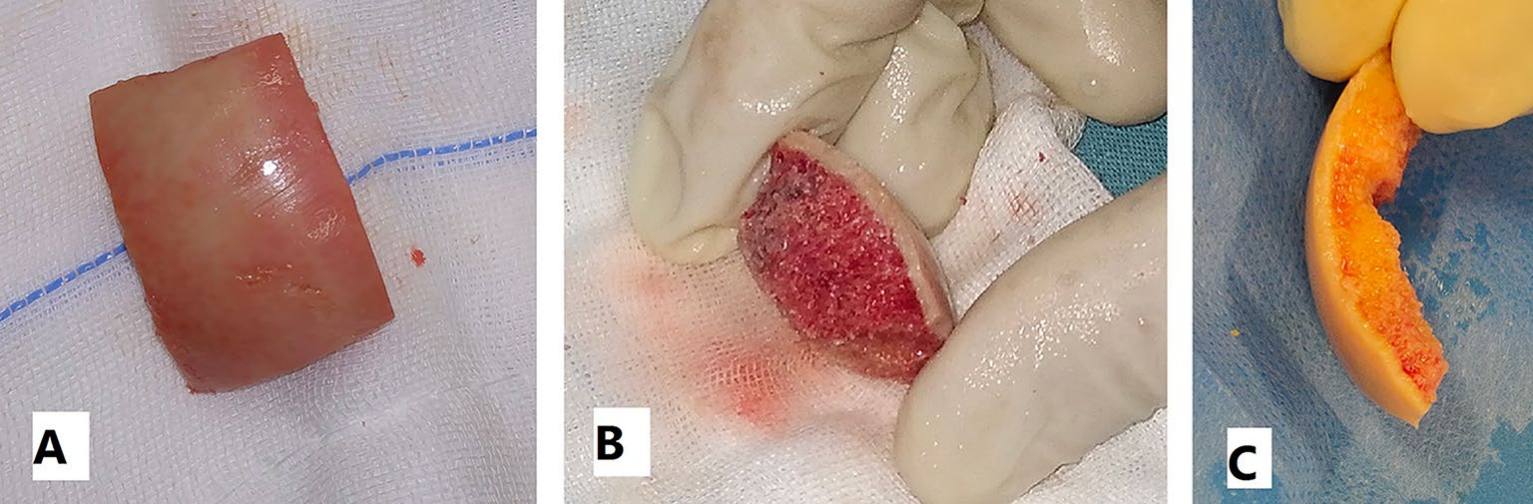
**A** and **B**: Subchondral bone was cut to match the same depth of the recipient defect. **C**: The graft was washed with saline to remove the marrow elements

**Fig. 3:**
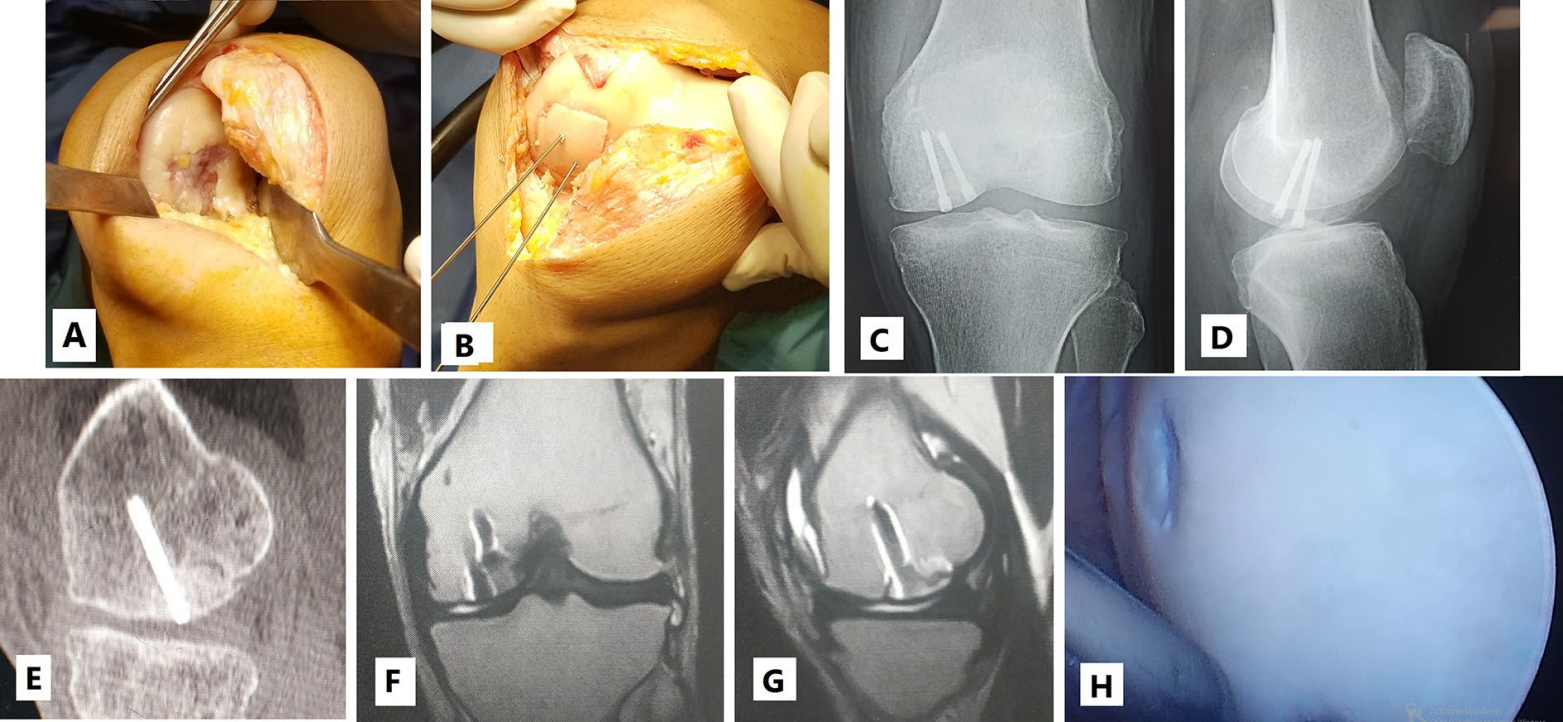
45 years old male presented 3 weeks after left knee trauma. **A**: Intraoperative image showing uncontained defect in the medial femoral condyle measuring 32 mm * 19 mm. **B**: Shell osteochondral allograft fixed by 2 headless compression screws. **C** and **D**: 3 months postoperative X-ray showing well contoured graft incorporation. **E**: 5 months postoperative CT scan showing well contoured graft incorporation. **F** and **G**: 5 months postoperative MRI showing 75–100% cartilage fill with good contour. **H**: 5 months postoperative second look arthroscopy showing intact hyaline cartilage

Knee arthroscopy was done in all cases to confirm cartilage defect size and location and examine the knee ligaments and menisci. Any loose bodies were removed. Stability of osteochondral fragments and subchondral bone were assessed during arthroscopy. Arthrotomy was done according to the location of the defect. The lesion size was measured after debridement of unstable cartilage flaps and lesion edges. One of two main techniques was used in our cases according to the lesion size. We used the shell technique if the defect size was more than 4 cm^2^ and the dowel technique if the lesion size was less than 4 cm^2^. In the shell technique, cartilage lesion edges were trimmed to make a regular edge. Subchondral bone was cut to the depth of 6–8 mm by a burr (Fig. 1). A piece of sterile paper was used as a template and cut to match the defect dimensions. The same surface area was cut from the femoral head. In order to avoid mismatch of the graft contour, we harvested the grafts from large femoral heads of male donors. We also cut the graft from the widest suprafoveal area of the femoral head. Subchondral bone was cut to match the same depth of the recipient defect. The graft was washed with saline to remove the marrow elements (Fig. 2). Then the graft was fixed using 2.0 mm and 2.7 mm Mini screws (Fig. 6) or 2.4 mm and 3.0 mm headless compression screws (Figs. 3, 9 and 10) (Synthes USA, Paoli, PA).

**Fig. 4:**
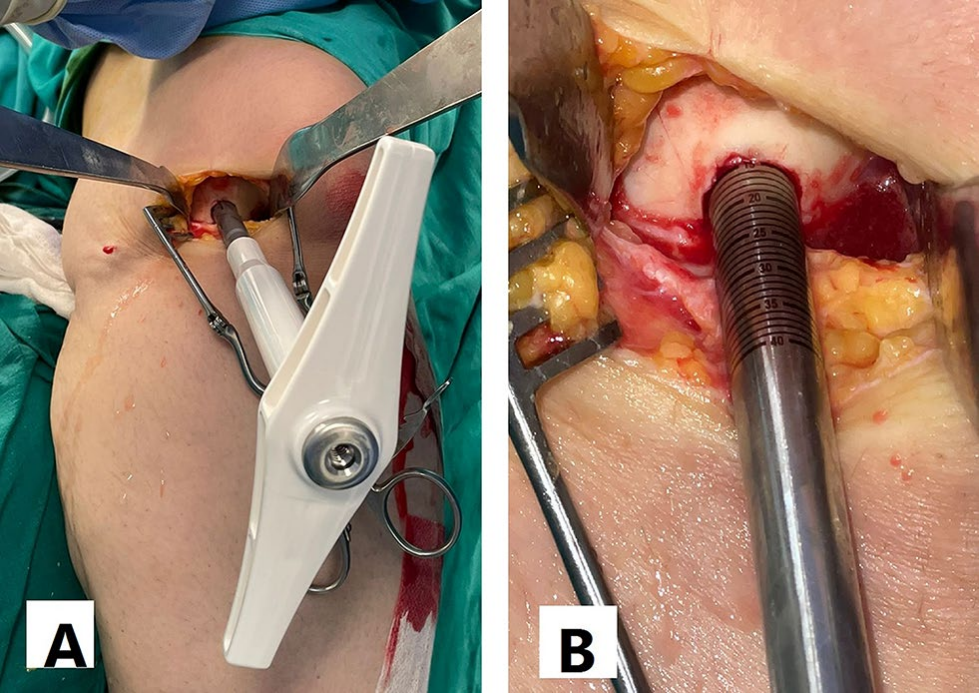
**A**: The lesion was cut by the recipient dowel knife to a depth of 6 to 15 mm. **B**: A system tool was used to assess the cut dowel depth

**Fig. 5:**
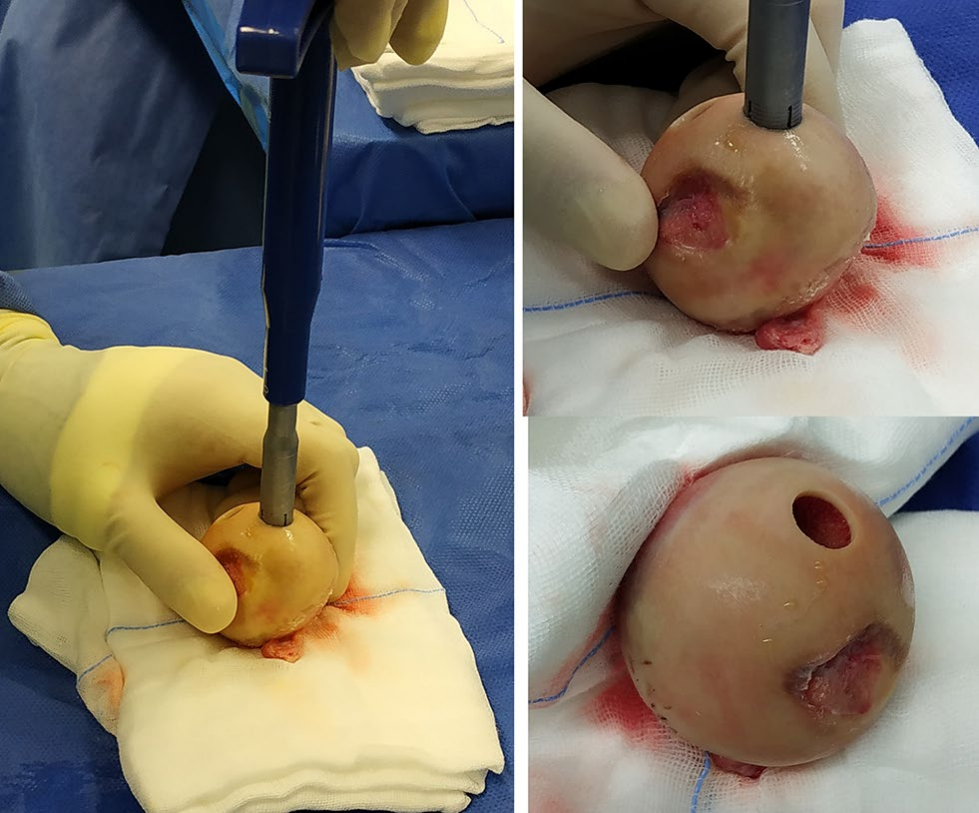
The femoral head was cut by the donor site dowel knife into a depth of one millimeter more than the recipient depth

**Fig. 6:**
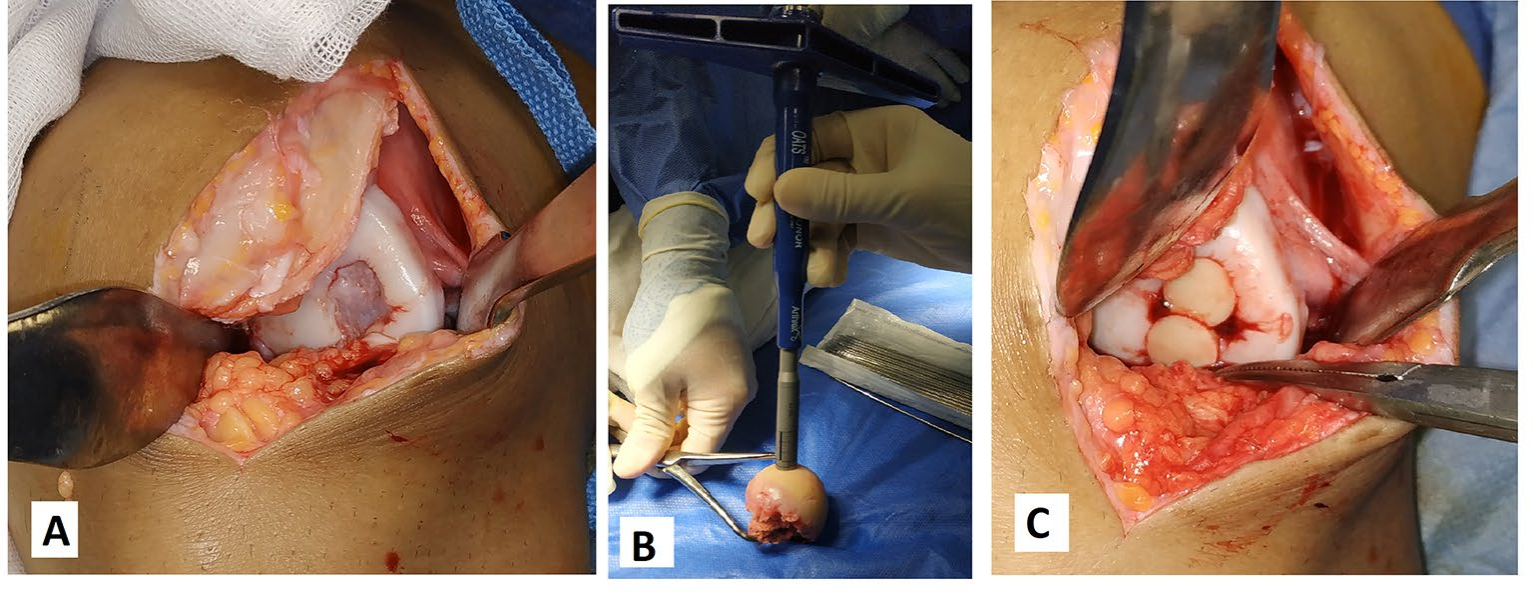
22 years old male Left knee. **A**: osteochondral defect lateral femoral condyle measuring 18 mm by 12 mm. **B**: 10 mm dowel donor site knife used on the femoral head. **C**: Two overlapping osteochondral allografts using the dowel technique

Alternatively, we used the dowel technique if the defect size was less than 4 cm^2^. It is difficult to restore a regular stable articular surface with too many dowels in lesions larger than 4 cm^2^ [10]. The use of multiple dowels in lesions larger than 4 cm^2^ is associated with fibrous tissue formation and fissuring between the grafts with poor outcomes [26, 27]. We harvested single or multiple dowels according to the lesion size using 8–10 mm Arthrex OATS system (Arthrex OATS system, Naples, FL, USA). First, the lesion was cut using the recipient dowel knife to a depth of 6 to 15 mm according to the status of subchondral bone (Fig. 4). Then the femoral head was cut using the donor site dowel knife to a depth of one millimeter more than the recipient depth (Fig. 5). The graft was washed with saline to remove the marrow elements. The graft was press-fit into the prepared recipient site. The press-fit was found to be secure enough precluding any other additional fixation. The same sequence was repeated for additional dowels (Fig. 6).

The same post-operative protocol was used in both groups. Early unrestricted passive range of motion was allowed from postoperative day one. Patients were kept non weight bearing until standard radiographs demonstrated graft incorporation; this ranged from 6 to 12 weeks according to graft size.

### Clinical assessment

All 15 patients were assessed using 2 subjective scoring systems: Lysholm and International Knee Documentation Committee (IKDC) scoring systems. Both scoring systems were filled by each patient preoperatively, 6 months postoperative and 12 months postoperative.

### Imaging assessment

Standard knee AP and lateral radiographs were performed immediately postoperative to assess the restoration of the femoral condyle contour. After 6 weeks, radiographs were performed every two weeks till graft incorporation. Follow up MR images were requested after 6 months postoperative. The Osteochondral Allograft MRI Scoring System (OCAMRISS) was used to assess the survival of the osteochondral allograft [28–33]. Scoring was performed according to the consensus of two experienced musculoskeletal radiologists. The OCAMRISS includes 5 primary cartilage features (cartilage signal of the graft relative to adjacent host cartilage, cartilage “fill” of the graft (percentage volume), cartilage edge integration at the host-graft junction, cartilage surface congruity of the graft and host-graft junction, calcified cartilage integrity of the graft), 4 primary bone features (subchondral bone plate congruity of graft and host-graft junction, subchondral bone marrow signal intensity of graft relative to epiphyseal bone, osseous integration at host-graft junction, and presence of cystic changes of graft and host-graft junction), and 4 ancillary features (opposing cartilage, meniscal tears, synovitis, and fat pad scarring).

### Statistical analysis

Analysis of data was done using SPSS program version 25 (IBM© Corp., Armonk, NY, USA). Quantitative data were presented as minimum, maximum, mean, SD and 95% confidence interval. Qualitative data were presented as count and percentage. Paired samples t test was used to compare quantitative data measured at two different time points for the same group. P value less than or equal to 0.05 was considered statistically significant.

## Results

Between April 2021 and June 2022, 19 patients who had preoperative diagnosis of full thickness cartilage injury were identified. Four patients were excluded from the study due to intraoperative arthroscopy findings. Two patients had healed injuries by white fibrocartilage. The third patient had kissing full thickness ulcer in the medial femoral condyle and medial tibial plateau and microfracture was done. The fourth patient had superficial cartilage injury for which ablation chondroplasty was done. Fifteen patients who received osteochondral allografts from the femoral heads of living donors were included in our prospective study (Table 1). Patients mean age was 33.8 years (± 5.7 years). There were twelve men and three women. Three patients had lesions in the lateral femoral condyle. The remaining twelve patients had lesions on the medial femoral condyle. The etiology of the lesions was traumatic in 2 patients, degenerative in 6 patients and advanced osteochondritis dessicans in 7 patients (Table 1). One patient had complex tear at the body of the medial meniscus involving less than 50% of the meniscal width. Arthroscopic partial menisectomy was done. On initial diagnostic evaluation, there were remnants of the injured cartilage discovered in traumatic and osteochondritis dessicans lesions. These were in the form of unstable cartilage flaps in 4 patients and loose bodies in 5 patients. There were no remnants of the injured cartilage in the 4 degenerative lesions. The subchondral bone affection varied in the 15 patients from being intact to deficient up to 5 mm depth. The average lesion size was 3.9 cm^2^ (± 1.1 cm^2^).

**Table 1:** Showing patients’ and grafts’ characteristics and outcomes

Id	Age	Lesion etiology	Lesion size Cm ^2^	Graft age (days)	Technique	Pre lysholm	6 MONTHS Post lysholm	12 MONTHS Post lysholm	Pre IKDC %	6 MONTHS Post IKDC %	12 MONTHS Post IKDC %	Follow up months
1	18	Traumatic	0.7	8	Dowel	66	71	79	58.6	69	70.1	25
2	21	Osteochondritis dessicans	3.6	10	Dowel	39	89	94	29.9	90.8	81.6	25
3	45	Traumatic	6.08	6	Shell	27	94	94	25.3	86.2	79.3	24
4	49	Degenerative	2	4	Dowel	52	76	86	16.1	60.9	71.3	23
5	39	Degenerative	5.8	12	Shell	31	62	69	26.4	52.9	50.6	22
6	52	Degenerative	2.1	7	Dowel	60	85	95	49.4	79.3	74.7	20
7	35	Osteochondritis dessicans	6.21	2	Shell	65	95	94	40.2	86.2	87.4	20
8	22	Osteochondritis dessicans	2.16	6	Dowel	70	90	95	59.8	87.4	85.1	19
9	31	Osteochondritis dessicans	1.8	1	Dowel	52	91	90	58.6	87.4	86.2	18
10	37	Degenerative	5.94	12	Shell	57	87	90	36.8	85.1	87.4	15
11	44	Degenerative	2	2	Dowel	47	85	87	34.5	72.4	79.3	14
12	24	Osteochondritis dessicans	7.14	2	Shell	81	87	95	60.9	75.9	77	13
13	45	Degenerative	5.8	3	Shell	23	67	82	17.2	57.5	69	13
14	25	Osteochondritis dessicans	5.22	3	Shell	23	84	88	13.8	60.9	75.9	12
15	20	Osteochondritis dessicans	2	4	Dowel	44	90	94	40.2	81.6	88.5	12

Our study included 15 grafts with average storage duration of 5.47 days (± 1.8 days).The mean donors’ age was 73.5 years (± 4.3 years). We used the free hand shell technique in 7 patients and the Dowel technique in 8 patients. In the Dowel technique, the average number of dowels was 2.13 per patient (± 0.54 dowels). There was a good restoration of the contour of the distal femoral condyle in all 15 cases.

### Clinical assessment

**Fig. 7:**
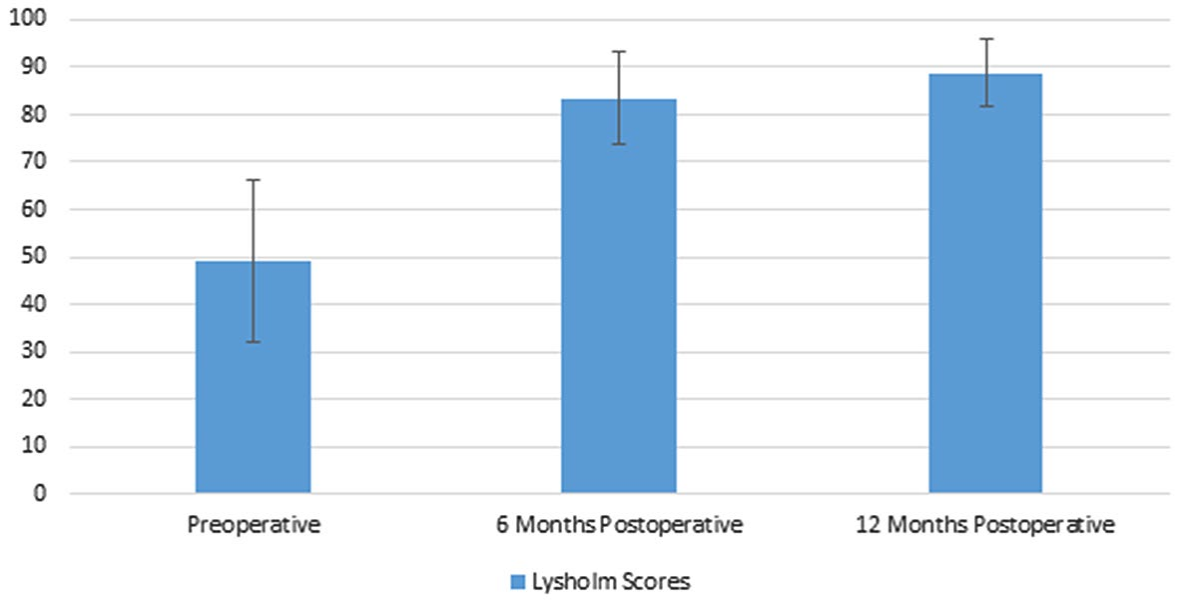
Preoperative and postoperative Lysholm scores

**Fig. 8:**
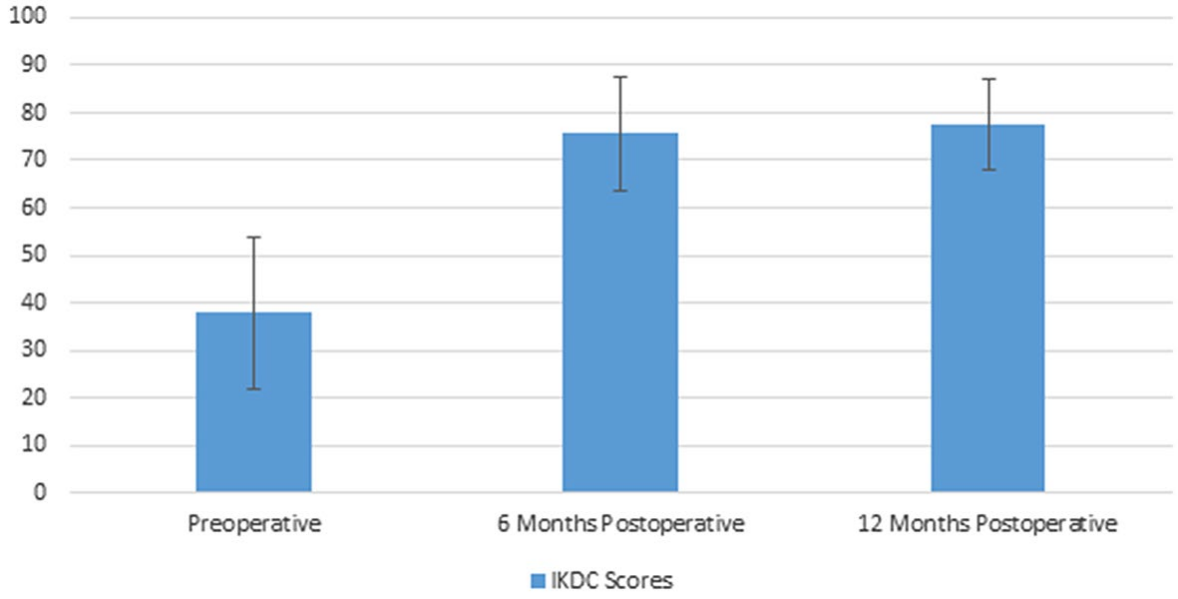
Preoperative and postoperative IKDC scores

Mean follow up was 18.3 months (± 2.37 months). Improvements in Lysholm and IKDC scores were statistically significant. The mean Lysholm scores (Fig. 7) improved from 49.1 (± 8.76) to 83.5 (± 4.85) and 88.8 (± 3.64) at 6 months postoperative and 12 months postoperative respectively (*P* < 0.001). The mean IKDC scores (Fig. 8) improved from 37.9% (± 8.12) to 75.6% (± 6.12) and 77.6% (± 4.86) at 6 months postoperative and 12 months postoperative respectively (*P* < 0.001).

### Imaging assessment

**Fig. 9:**
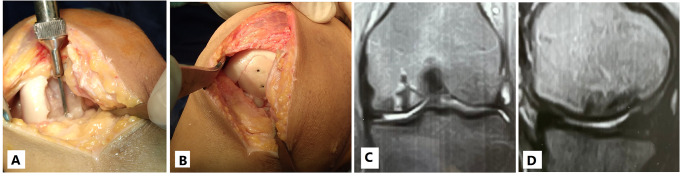
35 years old male right knee. **A**: Intraoperative image showing osteochondral defect of the medial femoral condyle measuring 27 mm * 23 mm. **B**: Shell osteochondral allograft fixed by two headless compression screws. C and D: 7 months postoperative MRI showing 75–100% cartilage fill with good contour

**Fig. 10:**
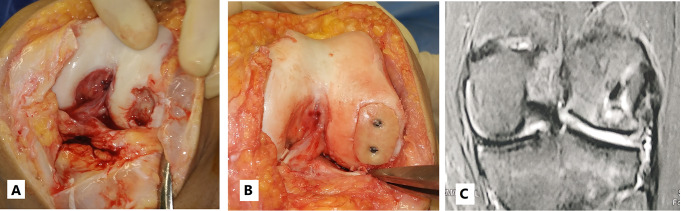
39 years old female Left knee. **A**: osteochondral defect lateral femoral condyle measuring 29 mm * 20 mm. **B**: osteochondral allograft using the shell technique fixed by two mini-screws. **C**: 8 months post-operative MRI showing 100% cartilage filling and good contour

Immediate postoperative radiography showed restoration of the distal femoral condyle contour. Delayed postoperative radiography showed graft incorporation in all patients (Fig. 3). The mean duration till radiographic graft incorporation was 8.4 weeks (± 1.29 weeks). Postoperative MR images were performed in 13 patients at an average of 6.8 months postoperative (± 0.88 months) (Figs. 9 and 10). Two patient refused to do postoperative MR imaging. They had painless full range of motion with good postoperative Lysholm and IKDC scores. A mean total OCAMRISS score was 3.4 (± 1.11). This total graft score included mean cartilage, bone and ancillary subscales of 1.2 (± 0.85), 1.8 (± 0.41) and 0.4 (± 0.33). The cartilage-fill item score for all 13 grafts was 76 to 100%.

### Arthroscopic assessment

**Fig. 11:**
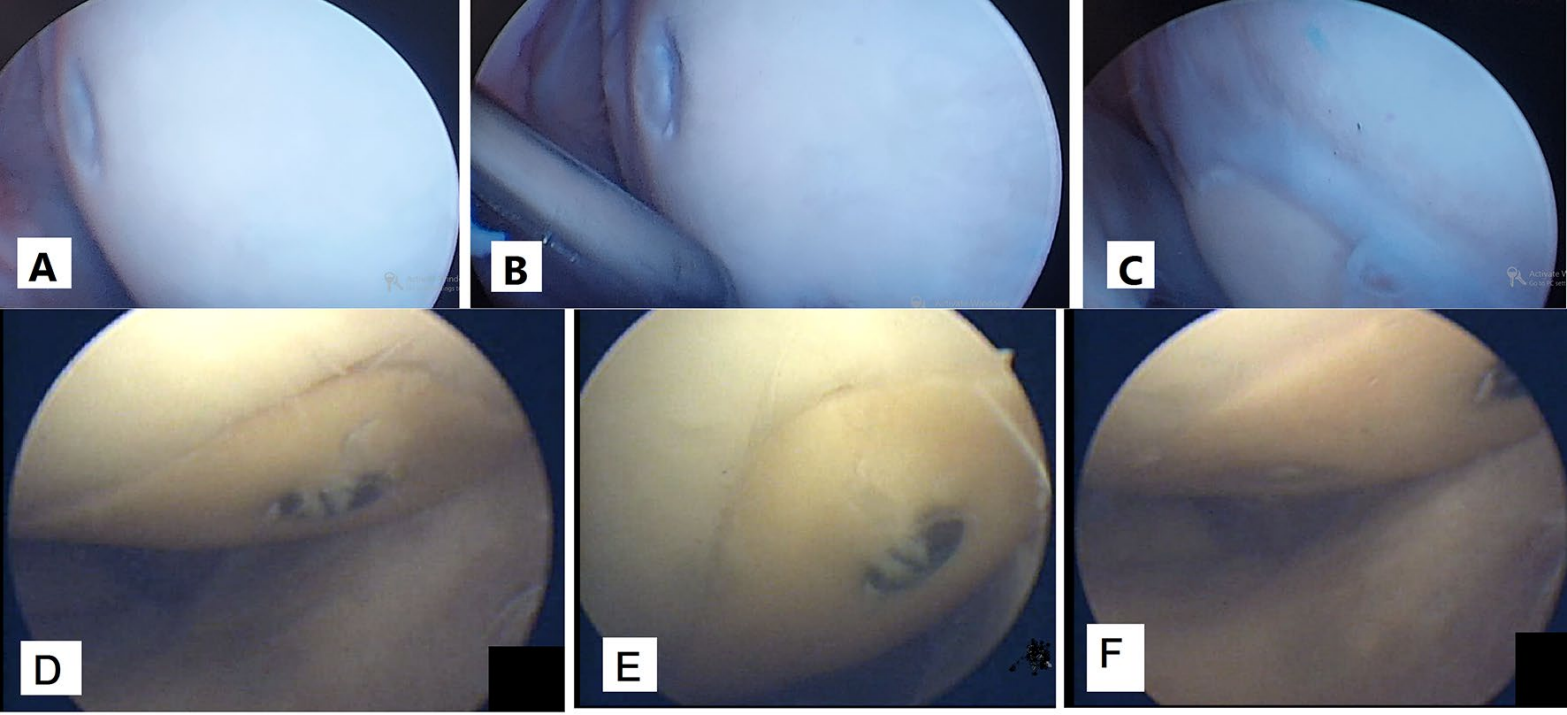
Second look arthroscopies of patients No. 3 (A, B and C) and No. 5 (D, E and F) showing intact allograft hyaline cartilage

A diagnostic second look arthroscopy was performed in four patients (Fig. 11). All four arthroscopies showed survival of intact regular articular cartilage. First patient diagnostic arthroscopy was performed 5 months postoperative early in the case series to confirm cartilage survival (Fig. 3; H). Arthroscopy was performed in two other patients to treat postoperative stiffness at 6 months postoperative. Arthroscopy was performed for the fourth patient at 3 month to remove a prominent screw.

### Complications

Out of the 15 patients only 3 cases (20%) had complications throughout the follow up period. Two cases had post-operative stiffness that was managed by arthroscopic adhesiolysis and mobilization under GA. The third case developed intra-articular screw prominence. It was managed by arthroscopic removal of the screw. There were no postoperative infections in all 15 patients.

## Discussion

Our study presents a new technique for fresh ostechondral allograft transplantation. The source of allografts is the non-arthritic femoral heads of patients undergoing hip replacement for femoral neck fractures. All fifteen patients had significant improvement in clinical knee scores. Postoperative radiography showed good restoration of the distal femoral condyle contour and graft incorporation. Postoperative MR images were assessed using OCAMRISS scoring system. MR images showed short term survival of regular articular cartilage. Second-look arthroscopy was done in four patients and showed intact allograft hyaline cartilage.

In our study, we achieved intraoperative and radiological restoration of the distal femoral condyle contour. Theoretically, the femoral head is more convex than the distal femoral condyles. This contour did not present a problem in small defects where we used multiple dowels. This is similar to the use of precut nonorthotopic grafts described by Jones et al. [34]. They used fresh precut osteochondral allograft cores taken from the lateral femoral condyle for a medial femoral condyle lesion. Fresh osteochondral allografts were also harvested from the distal femur and transplanted in the capitillum in cases of osteochondritis dessicans [35, 36]. Osteochondral autografts from the knee have been taken for a long time to treat osteochondral lesions of the talus [37]. In large defects where we used the shell technique, two defects measured 27 mm * 23 mm and 32*19 mm. They both were located in the lateral half of the medial femoral condyle weight bearing area. We achieved a good intraoperative and radiological restoration of the contour. This finding could be attributed to using larger femoral heads of male donors. We also harvested the graft from the widest suprafoveal part of the femoral head. Despite this, we think we may face a challenge with the contour in larger defects.

Comparison of our results with other studies in the literature could be difficult. We do not know about other clinical studies that used fresh living donor osteochondral allograft for restoration of knee full thickness cartilage defects. However in a histological study about osteochondral grafts from living donor knee arthroplasty resections, Hevesi et al [19] reported satisfactory chondrocyte viability and histologic quality compared to postmortem allografts. They mentioned that, the main limitation of allografts from being the gold standard in cartilage defects management is limited resources. Fresh osteochondral allografts are currently obtained from young deceased donors. Being young, the unexpected death of donors adds to the complexity of scheduling. This leads to difficult patient scheduling due absence of permanent stable resource. Hevesi et al. suggested the use of osteochondral allografts from the non-arthritic compartments of patients undergoing total knee replacement. They reported satisfactory chondrocyte viability and histology compared to postmortem allografts. They mentioned several advantages of the graft being from a living donor. First, it provides additional stable source of osteochondral allografts. Second, chondrocytes viability could be better if the transplant could be done earlier than the standard postmortem allografts. However, they mentioned a main limitation; their donor population is a highly selected subset of the total knee arthroplasty population. We think their technique may face another limitation if applied clinically. The thin total knee cuts will lead to a limited thickness of the subchondral bone allograft.

Long term outcomes of fresh osteochonral transplantation using allografts from deceased donors are reported in multiple studies. The allografts can survive with long term improvements in knee scores and functional outcomes reported up to 13 years [8, 10, 16]. The incidence of postoperative infections is low and ranges from 0.9 to 3.3% [38, 39].

There were no postoperative infections in all 15 cases. The femoral heads from living donors have lower risk of infection than postmortem allografts. This could be explained by the fact that arthroplasty patients must have prophylactic antibiotics, leading to a very low risk of hematogenous contamination by high virulence organisms. Also the graft harvest occurs exclusively in the operating theatre. There is only a low a risk of superficial contamination by the flora dispersed by the operating personnel [40]. In contrast, the postmortem graft harvest takes place in the operating room only in 33% of the grafts. The remaining 67% of the graft harvest occurs in the hospital morgue, coroner’s facility or the tissue bank facility [17]. This can lead to superficial contamination of the graft. Also there is a risk of postmortem septicemia that results from postmortem break in the gut barrier. This can lead to hematogenous contamination of the graft [40].

Regarding the postoperative MRI assessment, the mean total OCAMRISS score was 3.4 (± 1.11). This is comparable to the results published by Ackermann et al. [32] about a 6-month postoperative MRI osteochondral allograft evaluation, which showed a mean total OCAMRISS score of 3.9 ± 2.

This study has three main limitations. First, the short term follow up, especially that there is a theoretical concern regarding the survival of chondrocytes. Although we harvested the femoral head from non-arthritic donors, arthroplasty for femoral neck fractures is mainly done in the geriatric population. Despite good clinical and imaging short term results, long term survival is a major concern. However, the main concern in this study is to assess clinical outcomes, graft incorporation and cartilage survival which can be assessed in 6 months. Second limitation, the sample size is limited. We recommend further studies with larger sample size to assess the long term results of this technique. Third limitation, we did not perform histological analysis to assess the viability of chondrocytes at the time of transplantation.

This technique can have future implications regarding the fresh osteochondral allograft availability. Despite the increase in tissue banks, they are not available in all countries due to legislation issues. Even if tissue banks are available, the number of osteochondral allografts is still limited due to logistic scheduling challenges as they are obtained from young deceased donors. Our technique will provide an additional stable source of grafts which may expand the indications for osteochondral allografts and facilitate the preoperative scheduling. This will also provide a new source for fresh osteochondral allografts in countries who lack postmortem tissue banks. This is especially important in patients with large or uncontained defects for whom the autograft dowels is not a valid option.

## Conclusion

Femoral heads from living donors is a valid source of osteochondral allograft transplantation for knee full-thickness articular cartilage lesions. This technique showed good clinical and radiological short term results.
